# An Integrated Mass-Spectrometry Pipeline Identifies Novel Protein Coding-Regions in the Human Genome

**DOI:** 10.1371/journal.pone.0008949

**Published:** 2010-01-28

**Authors:** Danny A. Bitton, Duncan L. Smith, Yvonne Connolly, Paul J. Scutt, Crispin J. Miller

**Affiliations:** 1 Applied Computational Biology and Bioinformatics Group, Cancer Research UK, Paterson Institute for Cancer Research, The University of Manchester, Manchester, United Kingdom; 2 Biological Mass Spectrometry Facility, Cancer Research UK, Paterson Institute for Cancer Research, The University of Manchester, Manchester, United Kingdom; Texas A&M University, United States of America

## Abstract

**Background:**

Most protein mass spectrometry (MS) experiments rely on searches against a database of known or predicted proteins, limiting their ability as a gene discovery tool.

**Results:**

Using a search against an *in silico* translation of the entire human genome, combined with a series of annotation filters, we identified 346 putative novel peptides [False Discovery Rate (FDR)<5%] in a MS dataset derived from two human breast epithelial cell lines. A subset of these were then successfully validated by a different MS technique. Two of these correspond to novel isoforms of Heterogeneous Ribonuclear Proteins, while the rest correspond to novel loci.

**Conclusions:**

MS technology can be used for *ab initio* gene discovery in human data, which, since it is based on different underlying assumptions, identifies protein-coding genes not found by other techniques. As MS technology continues to evolve, such approaches will become increasingly powerful.

## Introduction

Since its release in 2001, the draft sequence of the human genome [1] has been revised numerous times and genome annotation continues to evolve [2]. Even so, the total number of genes is still unknown, and the estimated number (20,000–25,000) remains in dispute [3]–[7]. This lack of a definitive catalogue applies not only to genome databases, but also to the secondary protein and transcript databases upon which so many molecular biology assays are based. For example, mass spectrometry techniques that rely on a search against a database of known proteins will fail to identify previously unseen peptides, while the majority of microarrays, which are designed against a database of known or predicted transcripts, are unable to profile transcription that occurs outside those regions for which their probes were designed.

With the advent of next generation sequencing [8], [9], tiling [6], [10]–[13] and exon arrays [14], which feature probes targeting many more speculative areas of the genome [15], [16], numerous studies have found evidence for transcription outside known or predicted protein coding genes [6], [10]–[13], [17]. Much of this has been attributed to novel non-coding RNA, such as miRNAs [18], or to non-functional transcription, but, given the lack of a definitive catalogue of all human proteins, it is likely that at least some of this novel RNA is translated into previously unreported proteins [19].

High throughput tandem mass spectrometry (MS/MS) has become a favoured method for the identification of peptides and their cognate proteins in a complex protein mixture [20]–[25]. Such an approach normally leads to the production of thousands of spectra, each corresponding to the ion signature of a peptide, which are then identified using a database search algorithm such as Sequest [21], Mascot [24], or ProteinPilot [26]. These programs attempt to assign a peptide sequence to a spectrum, while ranking and scoring each assignment, and all assume that the peptide/protein exists in the database. This is a fundamental constraint that restricts the analysis to known and predicted proteins, and prohibits the discovery of novel coding regions.

A significant aspect of many proteomics experiments is the existence of ‘orphan’ peptides, those that have an experimental mass, but for which a sequence could not be assigned. A number of groups [27]–[34] have hypothesized that some of these may be due to the existence of novel protein sequences that are not currently represented in the databases, and have attempted to predict novel proteins by expanding the protein database used to identify proteins by tandem Mass Spectrometry (MS/MS) by translating the entire genome in all three forward and reverse reading frames [27], [28], [31]–[34]. The approach accepts the genetic code, but ignores the conventional signals of gene structure, such as initiation codon and known exon/intron boundaries. In so doing, more segments of the raw DNA sequence are represented by putative translation products, allowing greater coverage. However, two significant disadvantages are associated with this technique. Firstly, the extended search requires a much larger database of putative sequences, with a corresponding rise in the amount of time and space required to analyse the data, and secondly, the extended database will also contain a large number of spurious sequences, some of which may match the experimental data by chance [22], [31], [32], [35]. This magnifies the false positive rate, making it difficult to distinguish real matches from chance occurrences; already an issue with existing database searches.

Nevertheless, the approach has been applied successfully to plant [27], [28] and bacterial [34] genomes, allowing the detection of novel coding regions, the confirmation of gene predictions and the refinement of genome annotations. Recently, Tanner et al. [30] generated an expanded repertoire of predicted proteins using translations of EST and gene prediction data that were then used successfully to identify novel loci in human, while Menon et al. [29] were able to apply a similar approach in mouse. However, neither considered an unbiased full translation of the entire genome, in part because of the problem of controlling the False Discovery Rate (FDR) resulting from analyses against larger genomes.

**Figure 1:**
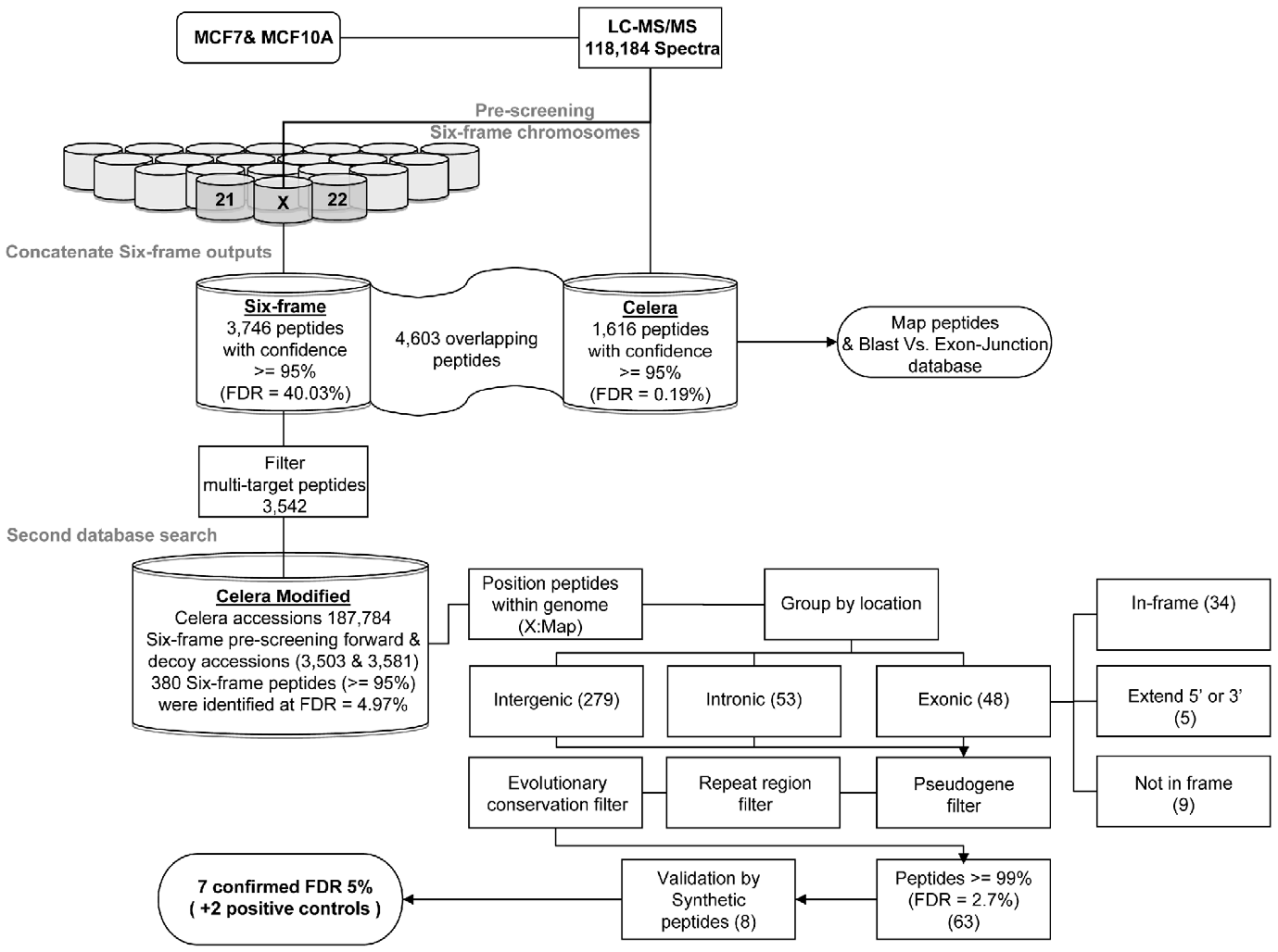
A pipeline to identify peptides originating from uncharacterised proteins using LC MS/MS data. Data are subjected first to identification using ProteinPilot and then filtered according to genome annotation. A subset of predicted novel peptides were then confirmed by addition MS/MS.

Here we describe a novel pipeline that employs a straightforward search against a six-frame translation of the human genome (Figure 1). We were able to identify and confirm experimentally that the pipeline does indeed identify novel proteins in high throughput MS/MS data. The pipeline uses a concatenated reverse database [36] to estimate the FDR and incorporates filtering steps that target pseudogenes, repeat elements and sequence conservation across genomes to find additional support for the assignments made by the database search algorithm.

Database searching was performed by first generating the full 6-frame translated database and an equivalent reverse decoy database [36]. Since it was not possible to concatenate these two databases and perform a single search, due to the amount of memory required by the software [30], we instead split the data by chromosome into 23 separate target and decoy databases. A series of pre-screening searches was then conducted on each individual database, to yield a set of target and decoy hits for each chromosome. In this way non-matching peptides were identified and eliminated from the analysis, making it possible to dramatically reduce the search space. Importantly, since decoy hits are also considered at this stage, it is possible to perform this data reduction step whilst preserving the information required for a reliable estimation of the FDR.

All hits resulting from this initial search were then combined with the Celera protein database to generate a single concatenated resource containing all possible spectrum-matching target and decoy peptides. All spectra were then searched for a second time against this reduced database in order to allow peptide assignments to be performed in the presence of other, competing, sequences. This is necessary since some spectra that match well in one chromosome may have a better match to a different sequence on another chromosome. These occurrences cannot be identified unless all candidate sequences are considered in a single batch. Similarly, some peptides that match to the decoy database may also have a better match to the target database. Again, this cannot be considered unless decoy and target sequences are searched together [36].

Candidate novel peptides resulting from this second search were then taken through for further analysis and validation, and the FDR estimated using the relative proportion of target and decoy hits, as previously described [36]–[38].

## Results

We evaluated the pipeline by applying it to a dataset produced using two human cell lines, MCF7 and MCF10A, a breast cancer and a non-tumourigenic epithelial cell line, respectively. Following sample preparation and processing, the resultant MS/MS data were searched once against the Celera protein database [39], and once against our novel six-frame translation database, using ProteinPilot.

### 6-Frame Proteogenomics Predicts Additional Protein Coding Loci in the Human Genome

Of the 8,349 putative hits identified following the pre-screening search, 4,603 were shared with the Celera database peptides (6,219), displaying 74% correspondence at the peptide level between the two database searches (Table 1). A total of 1,616 peptides found by the Celera search could not be identified using the initial six-frame searches, but, as expected, a considerable fraction of these (1,110) were found to span exon junctions (see methods), and a further 167 were assigned by the six-frame search, but were assigned a confidence of less than 95% by ProteinPilot. When these 1,277 peptides are accounted for, correspondence at the peptide level increases to 94.54%.

**Table 1:** Summary of the results obtained from LC-MS/MS analysis of the MCF7 and MCF10A cell lines.

Search/Level	Peptides > = 95% confidence	Peptides > = 99% confidence	Peptides > = 0% confidence	Spectra > = 0% confidence	% of spectra analysed
**Celera**	6,219 (FDR = 0.19%)	5,537 (FDR = 0.11%)	14,204	65,896	55.75
**Six-frame**	8,349 (FDR = 40.03%)	5,316 (FDR = 19.04%)	33,066	63,451	53.68
**Overlap**	4,603	4,048	6,983	50,694	N/A
**Celera unique**	1,616	1,489	7,221	15,202	N/A
**Six-frame unique**	3,746	1,268	26,083	12,757	N/A

Results of the initial pre-screening search in which all 118,184 spectra were searched against the individual chromosome specific six-frame databases and associated decoy databases. Data were also searched separately against the Celera database using ProteinPilot (ABI). For all searches FDR was estimated using the reverse decoy hits, as described in the methods.

A total of 3,746 matches with no high-confidence Celera equivalent were identified in the six-frame search. Of these, 119 matched to multiple sites in the putative translated genome, and 85 were found to have low confidence (<95%) matches in the Celera search. Both these sets of peptides were excluded from further analysis, leaving 3,542 peptide sequences (3,503 six-frame accessions) for further examination.

In the second database search, the full 118,184 MS/MS spectra were then compared to a single amalgamated database containing all Celera database entries (187,748), the 3,503 putative novel protein sequences, and all possible decoy hit accessions (3,581).

Following this search, 3,162/3,542 putative novel peptides were removed, leaving 380 peptides, identified with > = 95% confidence at a FDR of 4.97% (estimated using the reverse decoy hits [40]). Only these peptides were considered further.

These 380 peptides were then positioned relative to known genes using X:Map, a genome annotation database [41]. Peptides were classified as ‘intergenic’ (279 peptides), ‘intronic’ (53), and ‘exonic’ (48), based on their location relative to known protein coding features, as defined by Ensembl (version 47) [42]. Each exon is associated with a reading frame in which translation is expected to occur. Exonic peptides were further characterized as ‘in-frame’ when they occurred in the annotated reading frame (34), and ‘not in-frame’ when they matched the genome within an exon, but in a different reading frame to that annotated (9). Generally, ‘in frame’ peptides correspond to matches against known proteins, and are therefore of less interest when searching for novelty; they were not investigated further here. Finally, peptides found to extend the 3′ or 5′ ends (2 and 3, respectively) of an exon were labelled ‘exon-extending’ (Table 2).

**Table 2:** Pipeline predictions.

Peptide classification	Peptides > = 95% (2^nd^ search)	Pseudogene filter	Repeat filter	Conservation ΣR> = 0	> = 99% confidence cutoff	Peptides synthesized
**Intergenic**	279	269	157	101	56	7
**Intronic**	53	53	30	15	2	0
**Exonic ‘Not in Frame’**	9	8	7	3	3	0
**Exonic ‘Extending’**	5	4	3	3	2	1
**Total**	346	334	197	122	63	8

All 118,184 spectra were searched against a concatenated database comprising all Celera accessions, target and decoy hits from the pre-screening search (Table 1). Peptides that were uniquely identified by the six-frame search are referred as ‘orphan’ peptides. These peptides were classified according to their genomic position. ΣR – averaged conserved GERP score for the region from which the peptide originated. FDR was computed using the reverse decoy hits, as described in the methods.

Putative novel peptides were then subjected to a set of filters based on the location of repeat regions, pseudogenes and areas with high evolutionary conservation score (computed using GERP scoring across 10 species [43]). Peptides originating from more highly conserved regions that were annotated neither as repeat regions nor as pseudogenes were considered to be more likely to be biologically relevant (122 peptides).

### Confirmation of Proteogenomic Predictions by Comparison with Synthetic Peptide Spectra

When only 99% confidence peptides are considered, 63 are found by the pipeline (FDR: 2.7%). A subset of these (highest-confidence) peptides was selected for experimental validation. An underlying principle of protein MS is the assumption that under the same conditions the same peptide should fragment in a similar way, and thus yield a similar ion spectrum. The fragmentation pattern of a synthetic peptide should therefore be highly similar to that of a “real” peptide with the same sequence, making it possible to use synthetic peptides as a source of validatory spectra when seeking confirmation of a peptide assignment by MS/MS. Many of the spectra derived from complex mixtures feature ions that are not accounted for by the best sequence match. Often these are the result of the fragmentation of two different precursors simultaneously, leading to the production of chimeric spectra. Fragment ions that were carried over from a previous collision, background ions and/or inorganic compounds can also lead to additional peaks. When a single peptide is synthesized and analysed by MS/MS, its spectrum is less likely to contain these additional ions. In addition, post-translational modifications (PTMs) can also change the fragmentation pattern of a given peptide. 8 peptides for additional MS/MS validation, plus 2 positive controls, were chosen, by manual inspection of their spectra, to minimise these issues.

Synthetic peptides with the same sequences as the candidates were produced and subjected to MS/MS analysis in the usual way. 7 out of 8 of the synthetic peptides (plus both positive controls) were identified, at 99% confidence, with the same sequences as the “real” peptides, when searched using ProteinPilot against the augmented database. An additional comparison between the real and synthetic peptides, in which the number of common ions was used as a metric of similarity was also performed. FDR was determined empirically using a search of random, unrelated spectra (see methods), providing an estimate of the likelihood of a similar set of matches occurring by chance (Figure S1). At a 5% FDR, corresponding to a score threshold of 34.6, the same 7 sequences were found to be similar to their synthetic counterparts, along with both positive controls (Figures S1,S2).

**Figure 2:**
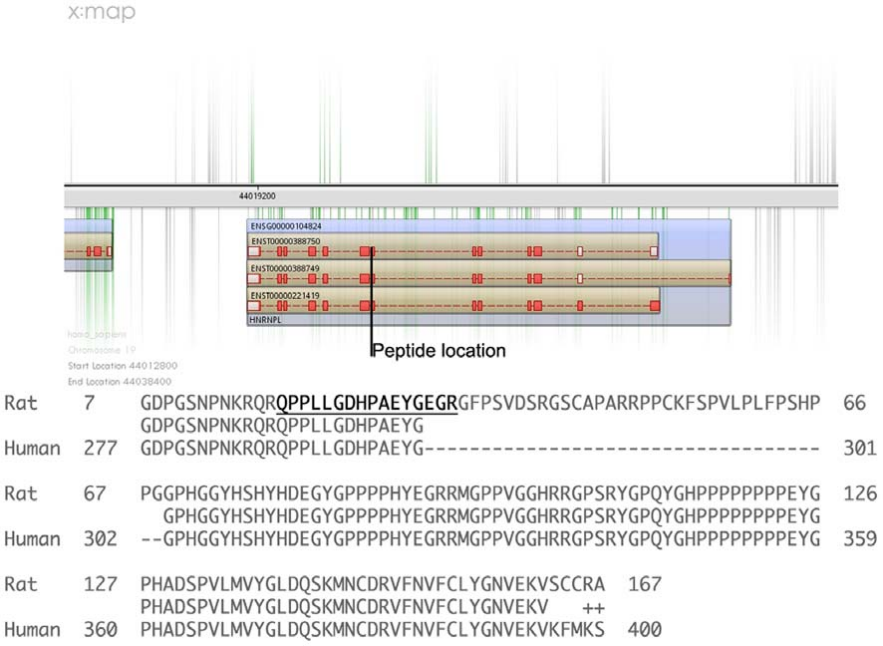
Location and nature of novel exon-3′ extending peptide in HNRNPL. **Top:** Location of peptide relative to exons. (Blue rectangle: gene; brown rectangles: transcripts; red/white rectangles: exons; red: coding, white: UTR). **Bottom:** alignment between NP_001128232.1 (hnRNPL isoform a, Rattus norvegicus) and HNRPL_HUMAN , showing location of the candidate peptide, and the retained intron found in the rat, but not the human, sequence.

Two of these 7 peptide sequences had high sequence similarity (BLAST [44]) to 2 distinct forms of Heterogeneous nuclear ribonucleoproteins [HNRNPL and HNRNPA1 like, chromosome 19 and 2, respectively (Table 3)]. HNRNPs play a major role in the packaging, processing, transporting and function of mRNA [45] as well as the modulation of splice site selection. One of these peptides (Peptide 3; Table 3 QPPLLGDHPAEYGEGR), also confirmed at the transcript level by RT-PCR (Figure S3), extends the 3′ end of exon 7 (ENSE00000704494) in HNRNPL, contributing an additional 3 amino acids to the protein sequence (Figure 2). Note that these additional 3 amino acids also provide the appropriate terminal arginine required for enzymatic cleavage by trypsin; the shorter form of the peptide would not have been identified. This exact peptide sequence was found to exist in both mouse and rat protein homologues, and alignments of these sequences found that the specified intron is retained in both organisms, encoding an additional 37 additional amino acids (Figure 2).

**Table 3:** Pipeline predictions confirmed by comparisons to spectra obtained from synthetic peptides.

	Peptide classification	Location	Sequence	Max common ions	Up/down MCF10A	Conservation ΣR
**1**	**Positive control**	Chr:8 (−1) 145066695–145066727	AGLVGPEFHEK	78	No	25.68
**2**	**Positive control**	Chr:17 (+1) 77639940–77639975	DNLEFFLAGIGR	137	Down	33.06
**3**	**Exonic extend**	Chr:19 (−1) 44022955–44023004	QPPLLGDHPAEYGEGR	126	No	64.54
**4**	**Intergenic**	Chr:3 (+1) 197481979–197482005	TQALVEILK	81	No	9.03
**5**	**Intergenic**	Chr:2 (+1) 194761171–194761215	SSGLYGGGGQSFDKP	56	Down	0
**6**	**Intergenic**	Chr:20 (−1) 46619533–46619600	SLATFQGQFNSWAGGPGSFVER	103	No	0
**7**	**Intergenic**	Chr:8 (+1) 21164240–21164278	TVGSRAATFVAGR	52	No	0
**8**	**Intergenic**	Chr:17 (+1) 74766590–74766619	GAVPASLAPK	47	Up	0
**9**	**Intergenic**	Chr:7 (+1) 43117591–43117632	GSVRKGLGTPSGIR	52	No	0

FDR calculated following 7,000 independent comparisons between a randomly chosen set of 10 different spectra (70,000 different spectra in total) and the synthetic spectra (8,672). RT PCR-reverse transcriptase PCR; Up/down regulation in MCF10A/MCF7 cell lines was determined using iTRAQ reporters in two experimental and a single control quantitation channels.

**Figure 3:**
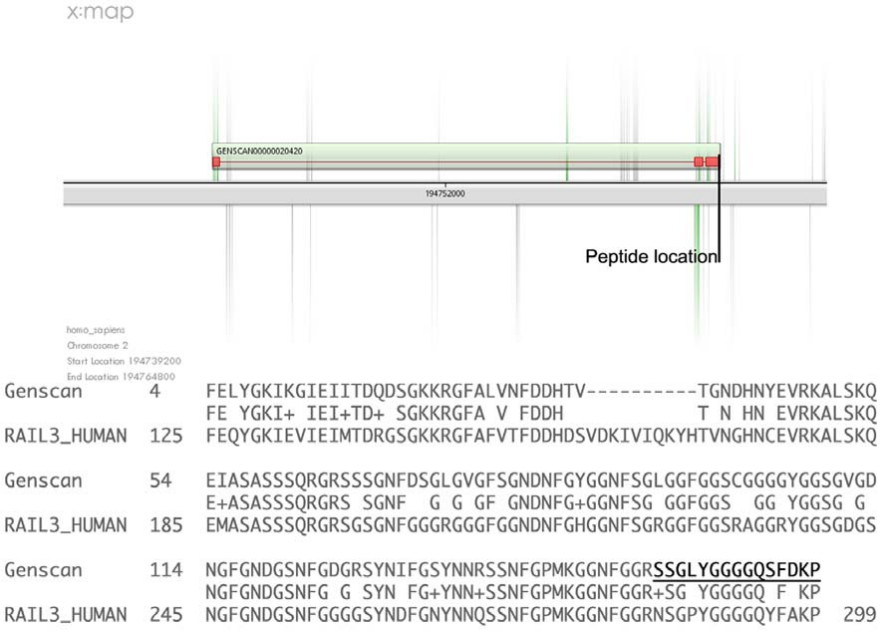
Location and nature of novel integenic peptide relative to Genscan prediction. **Top:** the peptide identified by the pipeline is classified as intronic, but is within the Genscan prediction GENSCAN00000020420. **Bottom:** the predicted protein is similar to hnRNPA1 (RA1L3_HUMAN; BLAST; Expect = 1e^−33^; 73% Identity).

The second peptide (Peptide 5; Table 3) fell within a region showing sequence similarity to HNRNPA1 (Table 2). This peptide prediction is located within a regional Genscan [46] (Figure 3), suggesting an open reading frame at this locus. A BLAST search and 3D homology modelling analysis predicts that the region encodes 271 amino acids (3 exons) that include the RNA binding domains necessary for a functional HNRNPA1 like protein (Figure S4), and transcript expression at this locus was again confirmed by RT-PCR. This second peptide was pseudo-tryptic (i.e. SSGLYGGGGQSFDKP), and the known HNRNPA1 protein was also identified in this dataset (ROA1_HUMAN), and a similar peptide sequence (SSGPYGGGGQYFAKPR) also contributed to the identification of ROA1_HUMAN. Given the similarity between both sequences it might appear at first sight as though the novel peptide might be erroneous. However, even though both sequences are very similar, the database searches are performed by comparing ion signatures, not amino acid sequences. It is thus important to consider differences at the spectrum level, rather than simply considering their alignments. A manual fragmentation simulation of both peptide sequences shows that they would produce very different fragmentation patterns (data not shown). In addition, both peptides were independently identified (by different spectra) in the database search, providing evidence in favour of both their existence in the proteome. It is unlikely that this peptide was simply an artefact.

The remaining 5 novel peptides (Table 3) displayed some weak similarity (expectation value>1) to human peptides, raising the possibility that some of the peptides could actually be explained by polymorphisms. However, in all cases the alignments either featured multiple mismatches, gaps, or lacked the necessary tryptic site(s) at the N or C termini. These differences would result in significantly different ion signatures, again making it unlikely that the matches are artefactual.

The lack of any strong similarity to known, well characterized, proteins (although there is cross-species sequence conservation at the DNA level) means that further characterization of these peptides would need to be performed experimentally. Seeking additional confirmation at the transcript level for these peptides is more challenging than for the two peptides described above, because the lack of additional data describing known or predicted gene-structures makes it difficult to position primers appropriately. Nevertheless, one of these sequences (Peptide 4; Table 3) was pursued further by RT-PCR (Figure S3), and transcription at that locus was again confirmed.

## Discussion

The incompleteness of current protein databases acts as a limiting factor when seeking novelty with MS/MS data. This can be minimized by a search against all possible protein products, generated by translating the entire genome in all reading frames, but is hampered by the corresponding increase in the False Discovery Rate, which makes it difficult to distinguish real events from chance occurrences [36]. The FDR is further magnified if it is necessary to search the target (and decoy) databases in batches, since the same spectrum can be assigned multiple times to different peptides. Here, for example, the FDR following the pre-screening stage was estimated at ∼40%, dropping to 5.0% following the second database search; a consequence of the removal of 90% of the candidate peptides by competition. Note that the shrinkage of the database is not the reason for this drop, since all possible decoy hits were included in the second search. This shows that it is possible to perform useful 6-frame translated searches against the entire human genome whilst controlling the FDR to reasonable levels; further supported by the successful confirmation of a subset of the candidate peptides by a different MS/MS approach.

We also considered using mRNA data to confirm the existence of transcription at each putative locus of translation. Two additional filters were applied based on Expressed Sequence Tag (EST) data and Affymetrix Exon arrays (data not shown). The former provides evidence that transcription has been previously observed at a given location, while the latter arrays feature many probesets targeting both EST predictions and those arising from *in silico* methods such as Genscan [46]. These can then be used to confirm transcription in mRNA samples paired with those subjected to MS/MS. When exon array and EST filtering were applied to the orphan peptide set, these two steps resulted in all but one of the orphan peptides being rejected, including all 8 that were taken through to experimental validation. This reflects the fact that both exon arrays and ESTs remain biased towards the better characterized (and, generally, protein coding) regions of the genome. As an extra validation step, we therefore used RT-PCR to confirm transcription for a subset of peptides, but this is not scalable across a large dataset. Clearly, technologies such as tiling arrays or next-generation sequencing may be used to provide a more global assessment of transcription [47], but since a substantial proportion of the human genome is now thought to be transcribed, these data may not prove to be particularly discriminatory. Additional resources might therefore be better directed at downstream validation, rather than further upstream filtering, or at increasing the coverage of the MS/MS data, since even if transcription is found at a given locus, this not conclusive evidence of translation.

Current mass spectrometry techniques are unable to resolve all proteins in a complex mixture, such as that arising from higher eukaryotic cells, and are biased towards high abundance peptides [48]. A recent meta-analysis of 2D proteomic data performed by Petrak *et al.* [49] revealed that similar lists of differentially expressed proteins are repeatedly reported by different researchers, regardless of the underlying experimental conditions, and similar concerns have also been voiced with respect to LC-MS/MS analyses, despite the greater proteome coverage that they offer [50], [51]. Thus, even though the dataset described here is likely to contain mostly high abundance, housekeeping proteins, we successfully identified and validated a novel isoform, a new gene paralogue and five putative novel coding regions, and predicted many more with high statistical significance. Given not only the stringency of the pipeline but also the cutoffs chosen for the validation by synthetic peptide, it is likely that the majority of these other peptides predicted by the analysis pipeline may also be real.

These results are also interesting because the first dogma of molecular biology – that DNA makes RNA makes protein – has dominated in the methodologies used to identify novel proteins, which are almost always inferred from known or predicted gene or transcript sequences. Advances in mass spectrometry offer an alternate route, in which novel genes can instead be inferred directly from experimental evidence at the peptide level. This relies on a different set of hypotheses and assumptions, and thus a different pattern of true and false positives. With stringent filters and appropriate validation, our methods successfully identify novel proteins that are not found using conventional techniques. As technology continues to improve, allowing the detection of lower-abundance peptides, such an approach will become increasingly powerful.

## Methods

### Protein Preparation and iTRAQ Labelling

2×10^6^ cells were washed with PBS, centrifuged at 500×g for 5 minutes and the dried pellet lysed in 0.5 M triethylammonium bicarbonate +0.05% (w/v) SDS. Protein was digested and iTRAQ labelled as described previously [52]. Briefly, 100 µg protein in 20 µl was reduced with 2 µl 50 mM tris-(2-carboxyethyl)-phosphine (TCEP) at 60°C for one hour and then alkylated with 1 µl of 200 mM methylmethanethiosulphate (MMTS) in isopropanol at room temperature for 10 minutes. Protein was digested by addition of 10 µl trypsin at 0.5 µg/µl and incubated at 37°C overnight. One unit of iTRAQ reagent (Applied Biosystems, Warrington, UK) was thawed and reconstituted in 70 µl of ethanol, with vortexing for 1 minute. The reagent solution was added to the digest and incubated at room temperature for one hour. Labelling reactions were then pooled prior to analysis. Two technical replicates were performed. MCF7 cells were labelled with 114 and 116 reporter ions, MCF10A with 115 and 117. Both cell lines were obtained from ATCC (LGC Standards, Middlesex, UK).

### Liquid Chromatography and Mass Spectrometry

Pooled labelled peptides were analysed as previously described [52]. Briefly, peptides were fractionated on an SCX cartridge (Applied Biosystems) in 10 mM K2HPO4 (pH 2.7)+20% ACN, with KCl concentration increasing in 50 mM steps from 50 mM to 500 mM. Peptide fractions were dried, and re-suspended in 240 µL 2% v/v ACN/0.1% v/v formic acid. 60 µL was loaded onto a 15cm reverse phase C18 column (75 µm i.d.) using an LC Packings UltiMate™ pump and peptides separated on a 80 min gradient from 5% to 40% v/v ACN/0.1% v/v formic acid on-line to a QSTAR® XL mass spectrometer.

### Six-Frame Translation Database

The complete human genomic sequence (Homo_sapiens.NCBI36.47) was translated in all reading frames. The translation of the genomic DNA started from the first, second and third nucleotide on each strand of each chromosome and ended whenever a stop codon was encountered. Triplets were translated according to the standard genetic code (IUPAC), to assign a one letter symbol for each amino acid and a ‘*’ symbol for a stop codon. A unique accession number that could be recognised by ProteinPilot (e.g. 1P_HUMAN) was assigned to each protein sequence and the genomic coordinates were recorded. Triplets containing ambiguity codes (i.e. ‘N’, ‘H’, ‘R’ etc.) were ignored, as were sequences shorter than six amino acids in length, and those which did not contain Arginine (R) or Lysine (K) (R and K not followed by Proline are the trypsin cleavage sites). Chromosome Y was not included (i.e. breast cancer cell lines). A total of 170,642,968 putative proteins were generated using Ensembl release 47.

### Relative Quantification and Peptide Assignments

iTRAQ data analysis and peptide/protein database searches were performed using ProteinPilot (version 2.0, Applied Biosystems, Warrington, UK). The uninterpreted spectra (118,184) were searched once against the human Celera protein database: human_KBMS5.0.20050302.fasta (187,748 proteins), and once against the six-frame database (one chromosome at a time). Only peptide matches with a confidence > = 95% were considered. The proteolytic cleavage was set to trypsin and the program was configured to report methylmethanethiosulphate (MMTS) as a fixed modification.

### Identification of Orphan Peptides

The list of Celera > = 95% confidence peptides that contributed to the protein identification (contribution>0) were compared to the complete list of the six-frame (regardless of their confidence/contribution), and vice versa. This accounts for cases when a given peptide sequence was assigned in both searches but the percentage confidence was different. Only exact matches were considered and isoforms, sequence differences between databases, polymorphism etc., were not included. A similar comparison based on the spectra rather than on peptides was also performed.

### Celera Peptide Mappings

The list of Celera peptides was locally BLAST searched (–M PAM30 –e 100 –W 2) against the human Ensembl [42] peptide database (Homo_sapiens.NCBI36.47.pep.all.fa) in order to retrieve Ensembl transcript IDs. This approach indirectly compares the Celera and the Ensembl databases. Minor discrepancies between the two databases therefore resulted in a small number of peptides not being mapped. The high e-value set for the BLAST search ensured that almost all possible hits were obtained. Nevertheless, only exact peptide matches of the same length as the query length were extracted. Finally, a BioPerl Ensembl API script [53] was used to pull out the peptides' genomic coordinates. For peptides located within exon-exon junctions, two sets of coordinates were retrieved. Similarly, a peptide sequence that exists in more than one place in the genome (e.g. shared between protein families), would also have more than one set of coordinates. These ‘multi-target’ peptides were excluded from further analysis.

### Mapping of the Six-Frame Peptides

In order to retrieve the exact genomic coordinates of the six-frame peptides, the parent putative proteins (ORFs) were retrieved from the six-frame database using fastacmd accompanied by the six-frame unique ID. Since the genomic coordinates were initially recorded during the database construction, it was possible to calculate the exact genomic position of the peptides simply by positioning the peptide sequences within their parent protein sequences. The BioPerl and Ensembl API script were also to confirm the exact location of the peptides, as described above. This accounts for cases when ProteinPilot assigned more than one unique accession to a given peptide (i.e. mapped to more than one place in the genome).

### The Exon Junction Database

The database was constructed using a list of all protein coding transcripts, as retrieved from X:Map. The exon sequences, along with the coordinates of their transcripts were retrieved. The 5′ and the 3′ sequence ends (54bp) of the exons were extracted, concatenated, shuffled and translated in three frames (the strand is known), so as to include all possible splice variants junctions. In cases where the exon ends were shorter than 54 bp the entire exon sequence was included. In addition, for 5′-terminal exons, only 3′ ends were used, whereas for 3′-terminal exons the 5′ ends were used.

### Analysis of Celera Unique Peptides

A considerable fraction of Celera peptides could not be identified by the search against the six-frame database. These were mapped back to their genomic coordinates, as before, while junction peptides were identified if two sets of coordinates were retrieved (in the same locus), or if they perfectly matched exon-junction database entries, following a BLAST search.

### Positioning & Grouping of Six-Frame Unique Peptides

‘Orphan’ peptides were positioned within the genome structure and classified according to their location using the exonmap library [41] in R/BioConductor. The exonmap R package supports a series of queries that enable direct mapping between probesets, exons, genes and transcripts to be made. The peptide coordinates were used to querying X:Map as follows: Firstly, each set of coordinates was used to search for a gene that may be found within its range. Secondly, each set of coordinates was used to search for an exon that may be found within its range. Then, the differences between the two search results and the initial list were identified, allowing peptides to be classified as exonic, intronic and intergenic. The ‘multi-targeted’ peptides were excluded from further analysis.

Exonic peptides were allocated to three subgroups (‘In-Frame’, ‘Not in frame’, ‘Extending’), based on whether they occur on the same frame as the exon from which they were originated (based on whether the peptide could be positioned within the translated transcript), or alternatively whether they extend their corresponding exon coordinates based comparison, Perl script).

### Pseudogene Filtering

The peptide's genomic sequences were retrieved using a BioPerl Ensembl API script. Thereafter, these sequences were BLAST searched against the manually curated human cDNA pseudogene database (Homo_sapiens_VEGA_jan_cdna_pseudo.fa, downloaded from ftp://ftp.sanger.ac.uk/pub/vega/). Only exact matches (peptides with 100% identity, same length, and same strand as the query sequence) were filtered out.

### Microarray Data Analysis

Briefly, 6 CEL files representing 6 chips (3 MCF7 and 3 MCF10A) were analysed. All analyses were performed using BioConductor/R [54] and the stored procedures found in the exonmap package , as described in [14]. Raw expression data were processed in R using the ‘affy’ BioConductor library. Expression summarisation was performed using RMA [55] with chip definitions supplied via a custom CDF file, as described in [14]. All data have been submitted to GEO (accession: GSE19154).

### EST Evidence

The peptide's genomic sequences were retrieved as above and BLAST searched against the human EST database (human_est.fasta, downloaded from ftp://ftp.ncbi.nlm.nih.gov/BLAST/db/FASTA/). Only exact matches (peptides with 100% identity, same length, and same strand as the query sequence) were retained.

### Repeat Region Filtering

The peptides' genomic coordinates were used to query the Ensembl API in order to exclude peptides that originated from repetitive regions.

### Conservation Across 10 Species

The peptides genomic coordinates were used to query the Ensembl API in order to assess the conservation of the peptides among different species (human, chimp, rhesus, cow, dog, mouse, rat, opossum, platypus, and chicken). Ensembl provides a nucleotide level GERP (Genomic Evolutionary Rate Profiling) scoring [43], that reflects the amount of inferred substitution, which in turn allows the identification of constrained elements. The substitution rate for each nucleotide was calculated as R = Σ(Expected-Observed) and a ΣR (sum of scores across the peptide) was calculated for each peptide and divided by its length. If scoring at that region was not available, R was reported as 0 and therefore ΣR = 0, while a positive ΣR should be expected in conserved regions and vice versa. In order to choose an appropriate cutoff value, 5,537 real exonic (Celera peptides with >99% confidence, FDR = 0.11%) and the ‘intergenic’ group of six-frame peptides (1,441 peptides with >99% confidence and FDR = 19.04%) were assessed and both distributions of ΣR values were plotted (all ΣR = 0 were removed). A non-parametric test (Wilcox rank sum test, wilcox.test command, R package) was performed to examine whether there was a difference between the two distributions, and a cutoff was chosen accordingly.

### Reverse Database and False Discovery Rate Calculations

Reverse database searches were performed using the PSPEP program [40] (Proteomics System Performance Evaluation Pipeline, ABI) that operates together with ProteinPilot. Since PSPEP estimates the false discovery rate within the concatenated database rather than the FDR solely within the target database, we estimated the FDRs under a given confidence threshold (95% & 99%) for each target database as discussed in [37], [38] FDR = (False positives/(False Positives + True Positives))*100 [37], [38].

### Second Database Search Against Modified Celera Database

A database search was performed (settings as above) against a modified Celera database that includes, all Celera database entries (187,748), the 3,542 putative novel protein sequences (3,503 accessions) and all decoy hits reported by ProteinPilot (regardless of their assigned confidence) following the pre-screening stage (3,581 accessions).

### Peptide Synthesis

Following re-identification of the putative novel peptides by the second database search, a manual examination of the corresponding 40 spectra was carried out. In total, 8 peptides were chosen along with 2 positive controls and synthesized (Eurogentec, minimum of 5mg of each with >70% purity). The peptides were iTRAQ labelled and subjected to LC-MS/MS analysis using the same settings as before, leading to the generation of 8,672 spectra.

### Comparisons of Fragmentation Patterns

Both the synthetic (8,672) and the original 118,184 spectra were converted to mgfs format (Mascot generic file) using ProteinPilot. Thereafter, the 10 original spectra (8 putative novel peptides and 2 positive controls) were extracted and compared to the synthetic spectra (4,531 scores). Pairs were scored by counting the number of common ions (excluding iTRAQ ions and potential ammonium ions, m/z>160). In order to generate random scores, 10 spectra (different from the original 10 peptide sequences) were randomly chosen from a pool of 118,105 spectra. This step was repeated 7,000 times (without replacement). Therefore, 70,000 random spectra were compared to the synthetic spectra (59.23% of the dataset), generating 518,112 random similarity scores. This was then used as a null distribution from which the FDR was calculated, as in [56].

### Homology Modelling and Structural Alignment

The protein sequence obtained from Genscan prediction (GENSCAN00000020420) was used for homology modelling in order to predict its 3D structure. This was performed using Swiss model [57] (automated mode settings), followed by structural alignment to its template using PyMOL [58].

### Reverse Transcription PCR

Total RNA was isolated from MCF7 and MCF10a cells using the Qiagen RNeasy kit (Qiagen, Sussex, UK). Genomic DNA was digested using RNase-free DNase (Qiagen, Sussex, UK). Reverse transcription was performed using Taqman reverse transcription reagents (Applied Biosystems, Foster City, CA, USA). The reaction included 1 µg total RNA, 2.5 µM random hexamers, Taqman RT buffer, 5.5 mM Magnesium Chloride, 500 µM each dNTP, 0.4 U/µl RNase inhibitor, 1.25 U/µl Multiscribe reverse transcriptase and RNase-free water to a total volume of 100 µl. The mixture was incubated at 25°C for 10 min, 48°C for 30 min and 95°C for 5 min.

PCR was performed using 1 µM each primer, 100 ng cDNA, 100 µM each dNTP, 2.5 U Taq polymerase, polymerase buffer and RNase-free water to a volume of 25 µl. Cycling conditions included denaturation at 94°C for 5 min, 35 cycles of 1) denaturation at 94°C for 30 sec 2) annealing at 60°C for 30 sec and 3) extension at 72°C for 1 min, finishing with a final extension of 72°C for 5 min. PCR fragments were resolved using the MultiNA Microchip Electrophoresis System (Shimadzu Biotech, Milton Keynes, UK).

Primer sequences: Control set (Left -TCCTCAAGTTTCCGCACAGT Right- GGCTGCCCATTTTGTATTGA, Product size - 82), Peptide 5 set (Left –TCAGTGGTCTTGGTGGCTTT, Right – CCACCATAGAGGCCAGAACT, Product size – 208), Peptide 3 set (Left – GCAGCAACCCCAACAAAC, Right – CCCTGCCCTCACCATATTCT, Product size – 75), Peptide 4 set (Left – CATTGGGGTGGGAAAAAGTT, Right – GGCCATTGTTGCACAGAGAG, Product size – 187).

## Supporting Information

Figure S1 Comparison between real and random spectra pairs. A) The distribution of the number of matching ions between random pairs (red, 518,112 scores) and “real” pairs (blue, 4,531 scores). The locations of the spectra identified in this assay are indicated by their reference number in table 3; two positive controls (1–2) and all putative novel peptides (3–9). B) The calculated FDR against the number of matching ions between spectra pairs.
(0.13 MB TIF)

Figure S2 Comparison between real and synthetic peptide sequences. Spectra from real (top) and synthetic peptides (bottom) for two positive controls (1–2) and all putative novel peptides (3–9)
(1.22 MB TIF)

Figure S3 Reverse Transcription PCR confirms transcript expression at loci corresponding to novel peptides. Primer sets specific to peptides 3, 4, 5, Table 3, were used to positively identify gene transcription by RT-PCR. The ribosomal protein L14 (RL14_HUMAN) was used as a positive control. Reverse transcription reactions were also performed in the absence of reverse transcriptase (RT) to confirm complete DNase I digestion. UM = Upper markers, LM = Lower markers. Expression for all targets was confirmed in MCF10A, while transcription for peptide 5 was inconclusive in MCF7.
(0.25 MB TIF)

Figure S4 3D structure of putative novel protein sequence. Protein sequence of GENSCAN00000020420 superimposed to crystal structure of UP1 complexed with D(TTAGGGTTAG(2PR)G) a human telemoeric repeat containing 2-AMINOPURINE (gold, PDB Accession 1u1r; X-RAY, Resolution: 1.80); Modelled by Swiss model server (Automated mode) [57]. Structures were superimposed using PyMOL.
(0.59 MB TIF)
