# Supplementary figures and images for: An Integrated Mass-Spectrometry Pipeline Identifies Novel Protein Coding-Regions in the Human Genome
Source: PLoS One. 2010 Jan 28;5(1):e8949. doi: 10.1371/journal.pone.0008949 (PMC2812506; doi:10.1371/journal.pone.0008949)

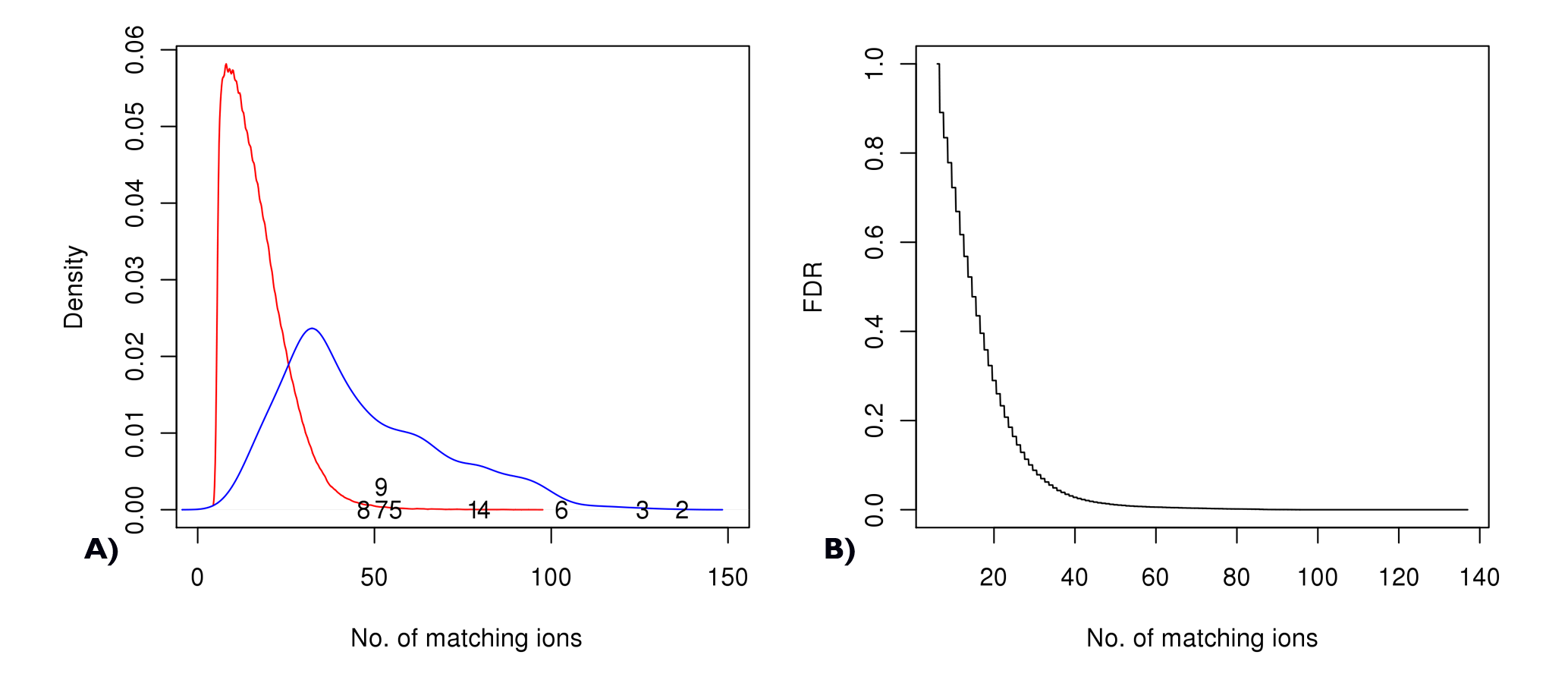

Supplement: Figure S1 — Comparison between real and random spectra pairs. A) The distribution of the number of matching ions between random pairs (red, 518,112 scores) and “real” pairs (blue, 4,531 scores). The locations of the spectra identified in this assay are indicated by their reference number in table 3; two positive controls (1–2) and all putative novel peptides (3–9). B) The calculated FDR against the number of matching ions between spectra pairs. (0.13 MB TIF) [file pone.0008949.s001.tif]

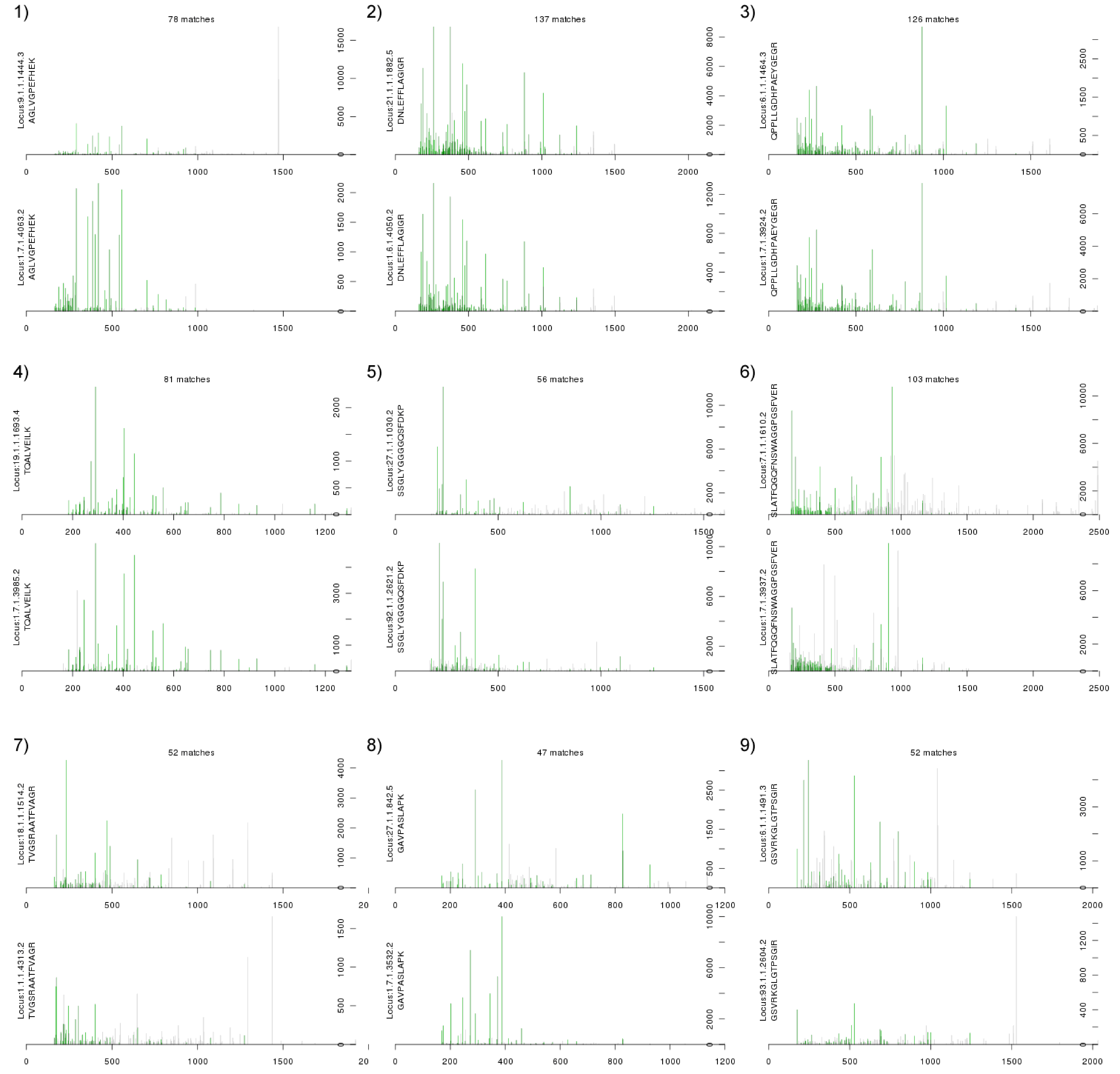

Supplement: Figure S2 — Comparison between real and synthetic peptide sequences. Spectra from real (top) and synthetic peptides (bottom) for two positive controls (1–2) and all putative novel peptides (3–9) (1.22 MB TIF) [file pone.0008949.s002.tif]

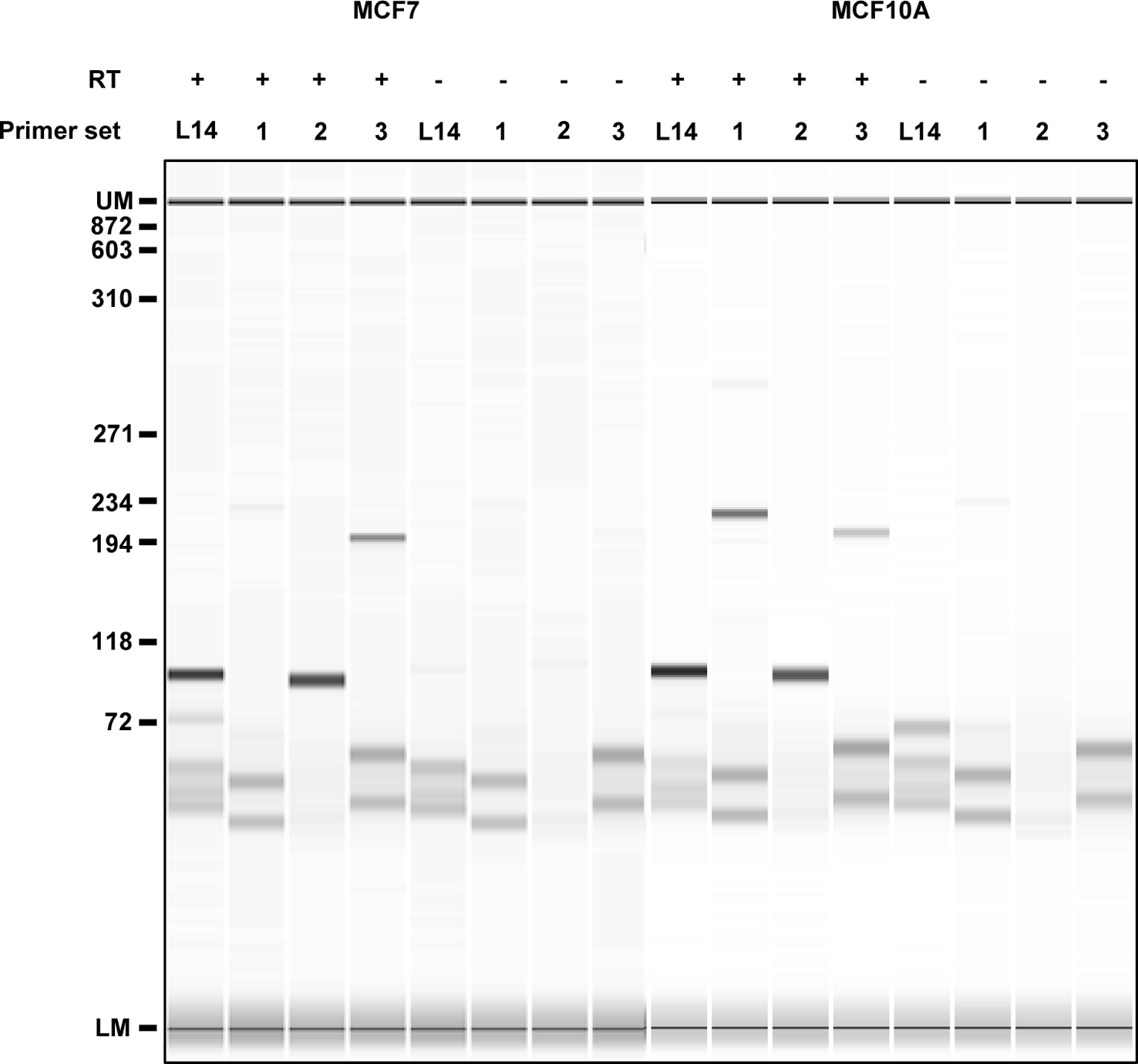

Supplement: Figure S3 — Reverse Transcription PCR confirms transcript expression at loci corresponding to novel peptides. Primer sets specific to peptides 3, 4, 5, Table 3, were used to positively identify gene transcription by RT-PCR. The ribosomal protein L14 (RL14_HUMAN) was used as a positive control. Reverse transcription reactions were also performed in the absence of reverse transcriptase (RT) to confirm complete DNase I digestion. UM = Upper markers, LM = Lower markers. Expression for all targets was confirmed in MCF10A, while transcription for peptide 5 was inconclusive in MCF7. (0.25 MB TIF) [file pone.0008949.s003.tif]

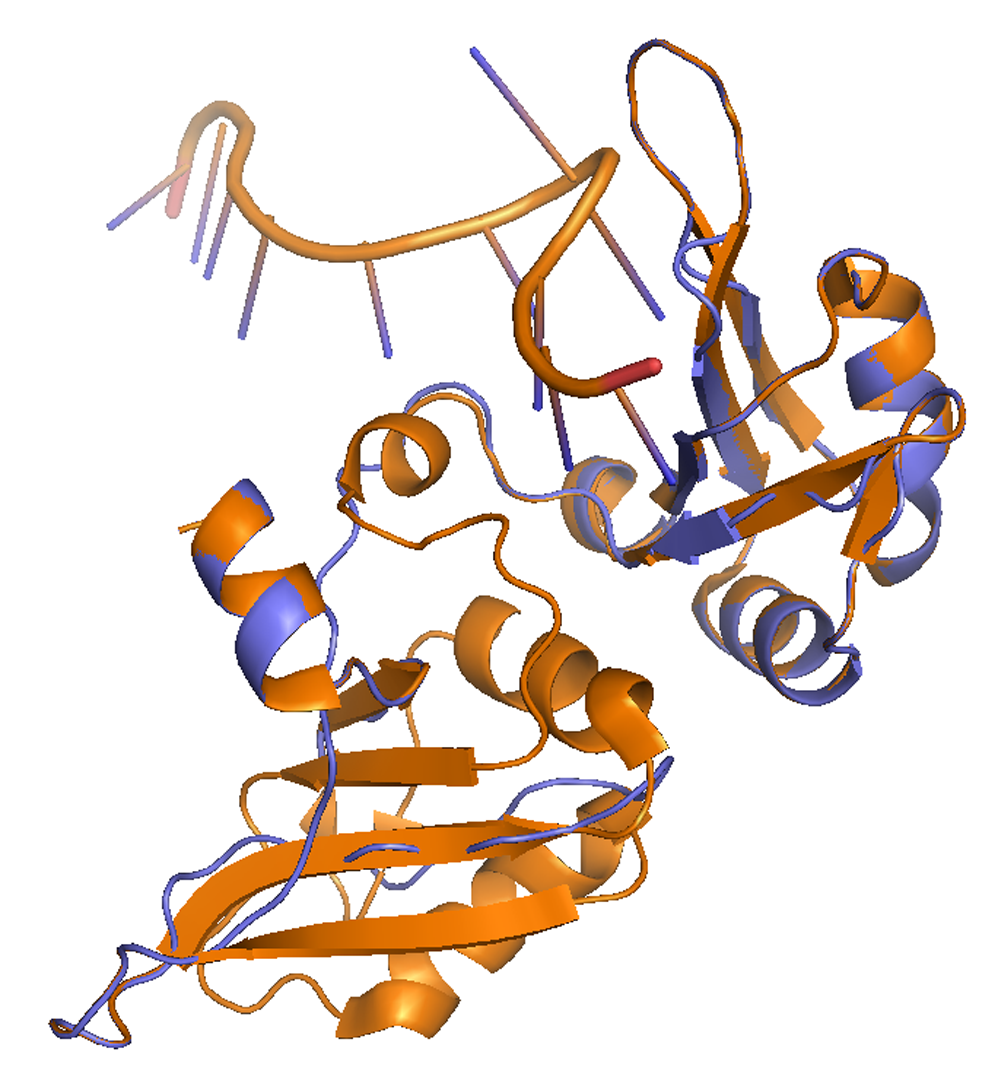

Supplement: Figure S4 — 3D structure of putative novel protein sequence. Protein sequence of GENSCAN00000020420 superimposed to crystal structure of UP1 complexed with D(TTAGGGTTAG(2PR)G) a human telemoeric repeat containing 2-AMINOPURINE (gold, PDB Accession 1u1r; X-RAY, Resolution: 1.80); Modelled by Swiss model server (Automated mode) [57]. Structures were superimposed using PyMOL. (0.59 MB TIF) [file pone.0008949.s004.tif]
